# A Reinforcement Sensor Embedded Vertical Handoff Controller for Vehicular Heterogeneous Wireless Networks

**DOI:** 10.3390/s131115026

**Published:** 2013-11-04

**Authors:** Limin Li, Yubin Xu, Boon-Hee Soong, Lin Ma

**Affiliations:** 1 CRC, School of Electronics and Information Engineering, Harbin Institute of Technology, Nan Gang District, Harbin 150001, China; E-Mail: malin@hit.edu.cn; 2 INFINITUS, School of Electrical and Electronic Engineering, Nanyang Technological University, Nanyang Avenue 639798, Singapore

**Keywords:** vehicular communication, heterogeneous network, vertical handoff, machine learning, automatic control

## Abstract

Vehicular communication platforms that provide real-time access to wireless networks have drawn more and more attention in recent years. IEEE 802.11p is the main radio access technology that supports communication for high mobility terminals, however, due to its limited coverage, IEEE 802.11p is usually deployed by coupling with cellular networks to achieve seamless mobility. In a heterogeneous cellular/802.11p network, vehicular communication is characterized by its short time span in association with a wireless local area network (WLAN). Moreover, for the media access control (MAC) scheme used for WLAN, the network throughput dramatically decreases with increasing user quantity. In response to these compelling problems, we propose a reinforcement sensor (RFS) embedded vertical handoff control strategy to support mobility management. The RFS has online learning capability and can provide optimal handoff decisions in an adaptive fashion without prior knowledge. The algorithm integrates considerations including vehicular mobility, traffic load, handoff latency, and network status. Simulation results verify that the proposed algorithm can adaptively adjust the handoff strategy, allowing users to stay connected to the best network. Furthermore, the algorithm can ensure that RSUs are adequate, thereby guaranteeing a high quality user experience.

## 1. Introduction

Vehicular communication platforms that provide real-time access to safety and entertainment services are attracting more and more attention. Infotainment applications are the key to opening up and expanding this new market because they can dramatically improve the driving experience. Traditional cellular networks can provide global wireless coverage, however, the IEEE 802.11p network, which is designed for high mobility terminals, represents a competitive alternative for vehicular communication [1]. This network mode can enhance data transmission, further enabling various infotainment applications. Additionally, it facilitates services for road safety and traffic management (*i.e.*, electronic tolls, traffic control, parking lot management) [2,3]. Therefore, roadside 802.11 access points, also called Roadside Units (RSUs), are important to supplement the cellular network for vehicular communication. In general, cellular systems perform better with high mobility services, even though these services provide lower data rates than WLANs. Heterogeneous wireless network technologies also help satisfy the diverse Quality of Service (QoS) demands from different users. It is evident that the inter-working and cooperation of both types of wireless networks will become an important issue in the future of vehicular communication.

Because different radio access technologies satisfy different QoS demands for different users, establishing a connection with the most suitable network under any given condition, which is called Always Best Connected (ABC), represents an interesting concept for increasing the diversity of wireless networks [4]. To realize this concept, heterogeneous mobility management has been comprehensively investigated. Vertical handoff (*i.e.*, the transfer of an active session across heterogeneous access networks) is an integral component of heterogeneous mobility management. When a user tries to switch to a more suitable network to optimize performance and monetary cost, a vertical handoff process will be initiated. This provides the capability of seamless communication and better QoS It is also the most relevant issue for meeting the goal of ABC heterogeneous networks. To implement this strategy, multiple radio interfaces need to be equipped on a terminal to support the vertical handoff. Such devices have been widely available in the market for several years. Vertical handoff can be categorized into two classes with respect to the types of networks involved in the handoff process. When the terminal switches from a large coverage network (e.g., cellular) to a small coverage network (e.g., WLAN), the process is describe as a Downward Vertical Handoff (DVH). When the terminal switches from a small coverage network to a large coverage network, the process is described as an Upward Vertical Handoff (UVH).

**Figure 1.**
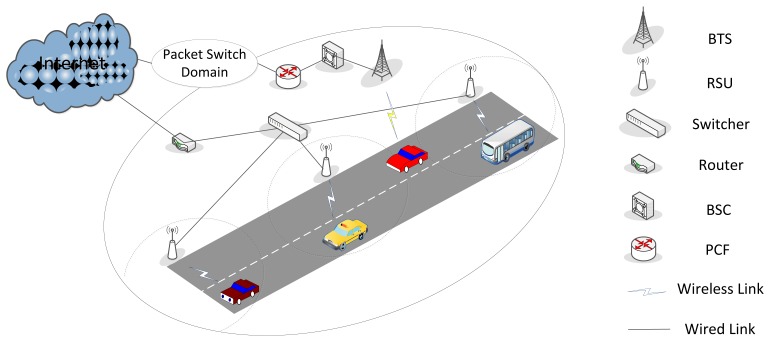
The reference scenario for the vehicular heterogeneous wireless network.

Vehicle to Infrastructure (V2I) and Vehicle to Vehicle (V2V) are the two enabling modes for vehicular communication by Intelligent Transportation System (ITS). In this work, we focus on a vehicular heterogeneous wireless network consisting of a global coverage cellular network complemented by a V2I communication mode, illustrated in Figure 1. The cellular network and the WLAN are loosely coupled. The main components of the cellular network are the Base Transceiver Station (BTS), Base Station Controller (BSC), Packet Control Function (PCF), and other components in the packet switch domain. Vehicles are equipped with On-Board Units (OBUs) that enable connections to RSUs. Because WLAN potentially provides better channel conditions with lower (or free) monetary costs, V2I communication is expected to improve QoS, enabling higher throughput and better balance for the network load.

However, vehicular communication is characterized by its short span of time in association with a RSU. Moreover, the MAC scheme for WLAN dramatically decreases network throughput with increasing user quantity. Therefore, a reasonable handoff control strategy should be devised to guarantee that the network works efficiently. As a counter-example, an inappropriate handoff controller may result in degeneration of the overall network performance when many vehicles try to switch to the same RSU. This will, in effect, lower the achievable data rate for WLAN relative to the cellular network due to increased congestion delays. Furthermore, this will decrease the Quality of Experience (QoE) for the user. For heterogeneous networks, many types of vertical handoff algorithms have been proposed to improve performance. A brief summary of these systems is provided below.

In 2004, McNair and Zhu proposed a comprehensive handoff algorithm that considered service type, monetary cost, network conditions, *etc.* [5]. In 2007, Lee *et al.* presented a movement-aware vertical handoff trigger scheme for WLAN/WiMAX heterogeneous networks that used an adaptive dwell timer to improve terminal connections [6]. In the same year, a SINR based vertical handoff algorithm was provided by Yang. This algorithm converted the SINR value from one network to an equivalent value for the target network to enable the handoff decision [7]. Soon after, Chang proposed an adaptive vertical handoff algorithm using predictive RSS. Simulation results were used to validate this algorithm, which was designed to reduce unnecessary handoff and improve dropping rate [8]. In 2011, Haider *et al.* used intelligent fusion of adaptive threshold, signal trend and dwell timer as the inputs of vertical handoff trigger algorithm and obtained a better result than many of traditional algorithms [9]. More recent studies were reported by Tabrizi in 2012. A Q-learning based strategy was adopted to achieve network selection with the goal of maximizing quality of experience. They found that the number of handoffs obtained through Q-learning algorithm can be significantly reduced compared to the optimum algorithm [10]. However, conventional handoff decision making algorithms are mainly based on the network status and the user's preference. They seldom consider the high mobility of a terminal, which is the most important characteristic of vehicular communication. When a handoff decision is executed, there can be trouble with switching to a WLAN network and then immediately switching back to the original cellular network. Therefore, vehicular mobility should be taken into account with respect to the feasibility of vertical handoff decisions.

Only a few studies have investigated the vertical handoff decision making mechanism using high mobility patterns for the users. A centralized Location Service Server (LSS) has been proposed for vertical handoff decisions [11]. Using this strategy, vehicles periodically report their current positions and receive information about potential nearby networks. A utility function is then used to calculate the satisfaction of the users with the available networks. This information is fed back to the LSS. Finally, the handoff decisions are provided by the LSS and reported back to the users. In [12], the authors proposed a distributed vertical handoff strategy for V2V and V2I communication and assessed the communication cost and transmission time. This work presented a heuristic analytical handoff strategy based on an ideal scenario, where the coverage radius of the WLAN and the data rate for the cellular network and the WLAN were fixed. Moreover, studies in [11,12] were based on the assumption that vehicles are equipped with GPS units that provide accurate positions at any time. This prerequisite might limit the feasibility and universality of the proposed strategies. In [13], the authors developed a vertical handoff control algorithm that took into account load balancing and battery lifetime maximization. In addition, they devised a route selection algorithm for forwarding data packets to the most appropriate attachment point for the same objective. In addition to these studies, an adaptive MAC scheme for WLAN has been proposed that uses traffic density data [14]. This algorithm can adjust the back off time for different traffic conditions and network conditions, achieving a lower collision and drop rate than traditional methods. Finally, a vehicular-IP MAC scheme that addresses the limitation of 802.11p standard for operation of IP applications was proposed in [15].

However, there are still many challenges for vertical handoff control in vehicular heterogeneous networks. To data, relatively little effort has been devoted to exploring vertical handoff strategies in realistic scenarios (e.g., multiple vehicles simultaneously accessing a RSU, and MAC layer adopts adaptive Modulation and Coding Scheme (MCS). Moreover, traditional vertical handoff control algorithms are mostly designer controlled. The handoff decisions are calculated by a predefined flow that integrates a number of metrics about network load, terminal status, and user's preference, *etc.* However, it is usually hard to establish a model for mapping from numerous related input parameters to arrive at an optimal decision. In particular, many metrics differ from case to case, such as channel parameter with respect to attenuation factor and standard deviation of shadow fading. These parameters vary based on the environment [16]. Furthermore, they need to be measured practically, which may be financially costly. These challenges severely restrict the feasibility and universality of traditional vertical handoff algorithms. This is the motivation for the work in this paper.

The key contribution of this paper is stated as follows: we propose an RFS embedded vertical handoff control strategy for vehicular heterogeneous networks. In contrast to traditional designer-controlled handoff algorithms, our proposed sensing system is based on robust control and employs adaptive real-time learning. Our system is objective-oriented, continuously exploring optimal solutions with respect to system performance. We apply our system for improving infotainment applications. These applications benefit from high throughput and are not sensitive to delay and jitter. The WLAN supplies relatively long service time by providing a higher data rate than the cellular network. For low traffic density, this handoff strategy can enlarge the valid area, allowing a higher number of users and increasing the accessing duration. The strategy can also shrink the valid area to enhance network utility and in avoid degeneration of the overall network performance. This strategy guarantees that RSUs can serve as high-throughput hotspots to supplement the cellular network. The algorithm integrates vehicular mobility, traffic load, handoff latency, network status, and link level data transmitting conditions. It can adjust the handoff policies for different input metrics, providing optimal handoff decisions without prior knowledge. It can also reduce the effort required to measure channel parameters such as attenuation factor and standard deviation of shadow fading, which are recognized as general requirements for traditional algorithms. Besides that, relative research verifies that channel parameters can be affected by temperature, humidity, and other environmental factors [17], decreasing the utility of handoff control strategy. This can be overcome by adaptively sensing environmental characteristics using our proposed algorithm.

Our paper is organized as follow: Section 2 provides a general description of the work, demonstrating the overall framework of the proposed vertical handoff controller. Section 3 presents the mathematical model for the RFS. Section 4 presents the simulation scenario and the numerical results. Section 4 also compares two basic types of handoff control methods, which are based on a threshold and a dwelling-timer. Finally, Section 5 provides our conclusions.

## 2. General Description

We focus on a typical vehicular communication scenario that is covered by both a cellular network and a WLAN, as depicted in Figure 1. The motivations for this choice are the widespread availability of dual-mode terminals for these technologies and the complementary characteristics of cellular networks and WLAN, where the cellular network provides a global coverage with relatively lower throughput and the WLAN provides support for higher data rates but with limited coverage. In the overlapping radio area of for the cellular network and the WLAN, the vehicle has the option of using either of them. Because the coverage range of WLAN is generally much smaller than the length of a major road, we confine the analysis in the spatial domain to a single direction of moving vehicles. We consider infotainment applications (*i.e.*, file transmitting, non-real time streaming) as the service type of communication. These services benefit from a high data rate, and they are not sensitive to delay or jitter. This is widely accepted as a necessary precondition for switching from cellular to WLAN. The proposed algorithm is expected to achieve autonomous control for the handoff requests to guarantee that vehicular terminals always connect to the best network with the highest individual throughput. Note that there are two modes for V2I communication allowed by 802.11p. Association or authentication is not required when the management information base (MIB) attribute “dot11OCBEnabled” is “true”. This access scheme benefits certain types of services that require low latency (e.g., safety related services). On the other hand, the shortcoming is that such stations are neither associated nor authenticated, and the authentication and data confidentiality cannot be guaranteed. The other mode is that, an on-board unit for which “dot11OCBEnabled” is “false” still needs to complete an association and authentication procedure, which are the same as in the 802.11 managed mode. In this paper, we adopt the second mode due to the fact that the proposal focuses on private data services, which are not safety related. Therefore, the access procedure is the same with 802.11 managed mode, for which a vertical handoff procedure always follows a considerable latency. To obtain relatively realistic simulation results, different data rates based on the channel condition, are adopted in the MAC layer of WLAN.

Q-learning is a classical reinforcement learning algorithm with outstanding self-adaptive capability that is used as the core component of the RFS. It can function for sensing and learning. Originally proposed by Watkins and Dayan [18], Q-learning has been widely explored in the field of automatic control. In the original literature, the overall framework of the learning procedure is demonstrated. Many componential functions of Q-learning (e.g., membership function and reward sensing function) are not specified in the original work. These issues are left up to scholars and engineers who want to develop the algorithm for specific problems. We propose a complete judging, sensing and learning procedure (detailed in Section 3). Meanwhile, to overcome the limitations of classical Q-learning (e.g., discrete state perception and discrete actions), a Neural Fuzzy Inference System (NFIS) is adopted to retain continuous perception of the state space. This algorithm infers the global policy, which is relative to the state, from local policies associated with each rule of the learner. With respect to machine learning, NFIS is embedded to introduce generalization in the state space and generate continuous actions. On the other hand, with respect to fuzzy logic's point, Q-learning is used to tune the fuzzy controller.

Our proposed strategy senses the feasibility of handoff decisions and explores optimal solutions for different traffic and channel conditions. It can be used as an independent unit in the RSU for handoff control. Therefore, we regard it as a generalized sensor, although it does not sense physical measurements. The proposed algorithm is considered to be a centralized handoff controller. It can be integrated as a module in the RSU. In contrast to distributed handoff controlling strategies, it avoids high latencies and loss of large numbers of packets during the handoff process [19]. Another advantage of its centralized structure is that it can guarantee network performance from an overall point of view, which cannot be achieved using a distributed structure. We assume the information needed by handoff control can be sent by periodically broadcast beacon messages. RFS is designed to work before the authentication process. If the handoff decision is “yes”, then the conventional authentication and association procedure will be triggered. On the other hand, if the handoff decision is “no”, then the request will be ignored and dropped. The OBU needs to wait after the interval of handoff request to initiate the next handoff request.

### 2.1. DVH Process

**Figure 2.**
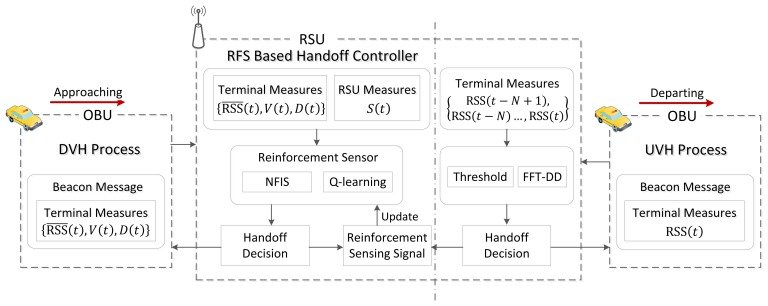
The architecture of the RFS embedded vertical handoff controller.

Figure 2 shows the approximate flow for the handoff processing based on RFS. In the left part of the figure, when an OBU approaches an RSU and receives the beacon signal, it will initiate the vertical handoff process. We assume the information needed by handoff control, including the measures of $\bar{\text{RSS}}(t)$, *V*(*t*), and *D*(*t*), can be sent by periodically broadcast beacon messages, where *t* is the time instant. A similar approach was used in the work of [12]. According to the protocol [1], “Vendor Specific” of beacon frame can be used to carry the needed information. Considering the potential range of the measurements, each of them can be recorded and transmitted by using 7 bits, which includes 128 levels. Therefore, the overhead for each beacon frame is 21 bits. Here we use the mean value of the RSS between the interval of current handoff request and the previous request. This can reduce the deviation caused by shadow fading according to the maximum likelihood estimation principle. Symbol *V* and *D* are the velocity of OBU and the data quantity to be transmitted, respectively. The handoff controller sends these measurements, along with *S*, which is the quantity of real time users, to the reinforcement sensor. RSS and *V* relate to the vehicular position and motion information. *D* reflects the necessity of vertical handoff. For services involving known data transmission (e.g., file downloading and video streaming), *D* is used as an input to avoid unnecessary handoffs. For example, an OBU can switch into WLAN for a better data rate, even though the session is already going to be finished. Note that the data to be transmitted cannot be obtained for every type of service. For some cases, this parameter is set as an infinite value. The data to be transmitted is then regarded as a large quantity by fuzzification function. It will not affect the judgment of the handoff decision. Based on the MAC scheme for 802.11p, the quantity of real-time users denoted by *S* directly reflects the current network performance. We take these metrics as the input for the vertical handoff decision making system.

The reinforcement sensor consists of Q-learning and NFIS, where Q-learning is used to achieve an adaptively sensing ability and NFIS is used to retain a continuous perception of the state space. A handoff decision will be given as the output of the RFS. The NFIS follow Takagi-Sugeno [20] structure, whereby each fuzzy rule is in the form of:

$$\begin{array}{c} \text{IF}\;\bar{\text{RSS}}(t)\;\text{is}\;M_{1,j_{1}}\;\text{AND}\;V(t)\;\text{is}\;M_{1,j_{2}}\;\text{AND}\;D(t)\;\text{is}\;M_{1,j_{3}}\;\text{AND}\;S(t)\;\text{is}\;M_{1,j_{4}}\\ \text{THEN}\;\text{handoff action is}\;A\;\text{with}\;Q, \end{array}$$

where ℳ*_i,ji_* is the linguistic *j_i_* th variable related to the *i*th input metric, and ℚ is the reinforcement sensing value that guides the self-learning of RFS. A detailed description is presented in Section 3.

### 2.2. UVH Process

When the vehicle is departing the RSU, the RSS becomes weak and the MCS level becomes low. As a result, the data rate will decline gradually. Because the vehicle has already associated with the RSU, the individual throughput is known. Therefore, the handoff strategy from the WLAN to the cellular network can be relatively simpler than the reverse procedure. To reduce the computational complexity, we apply an algorithm that integrates the threshold and Fast Fourier Transformation based Decay Detection (FFT-DD) [21], as depicted in the right part of Figure 2. FFT-DD is employed to determine the trend of the WLAN signal and identify the vehicle as it approaches and departs the RSU. For this purpose, we calculate the imaginary part of the fundamental component of the FFT for the past *Ω* WLAN signal samples, as shown in Equation (1):

(1) $$X(t)=\sum_{\omega =0}^{\Omega -1}\text{RSS}\left(t-\omega \right)\sin\left(\frac{-2\pi \omega }{\Omega }\right)$$

A positive value for *X*(*t*) indicates a rising signal trend, while a negative value reflects a decaying signal trend. The duration of the vehicle association with the RSU is relatively short. Therefore, we assume that the cellular data rate is steady during this period and is used it as a reference for the handoff judgment. The UVH process is initiated when the data rate is lower for WLAN than for cellular and the FFT-DD indicates that the RSS signal is decaying. When an OBU switches back to the cellular network, the backward propagation of the RFS is triggered. This procedure achieves the sensing and parametric learning functions. The handoff controller evaluates the complete handoff actions of the DVH and UVH. It then tunes the parameters for the RFS. The sensing and learning process can be switched off to reduce computational complexity when the handoff control system collects enough knowledge and achieves stability. The detailed scheme for the RFS is described in the next section. The pseudo-code for the proposed handoff control scheme is presented in Table 1.

### 3. Mathematical Model

**Figure 3.**
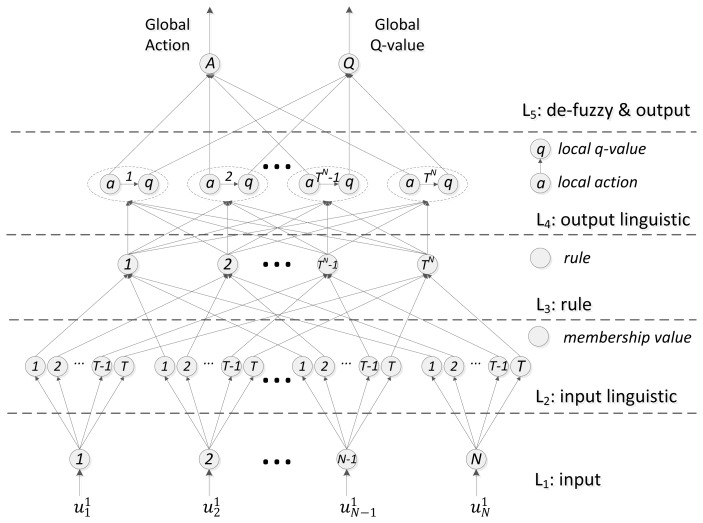
The topology of the reinforcement sensor.

The topology of the reinforcement sensor is shown in Figure 3. The neurons in the different layers achieve different functions. The only information available for learning is the system feedback, which is the reinforcement sensing signal according to the last action it performed in the previous state (e.g., switching out of the RSU). We use $u_{i}^{j}$ and $O_{i}^{j}$ to represent the input and output of the *i*th node in *j* th layer, respectively. To demonstrate the mathematical model in a universal manner, we include equations with unfixed dimensions for the input state vector (*N*) and membership function (*T*)

#### 3.1. Forward Propagation

*L_1_input layer*: This layer consists of *N* neurons, all of which transmit the input value directly by:

(2) $$O_{i}^{1}=u_{i}^{1}\;,\;\forall i\in \left\{1,2,\ldots ,N\right\}$$

We use $\bar{\text{RSS}}(t)$, *V*(*t*), *S*(*t*) and *D*(*t*) for the input state vector. Therefore the input can be represented by Equation (3):

(3) $${\text{U}}^{1}\left(t\right)=\left[u_{1}^{1}\left(t\right),u_{2}^{1}\left(t\right),u_{3}^{1}\left(t\right),u_{4}^{1}\left(t\right)\right]=\left[\bar{\text{RSS}}\left(t\right),V\left(t\right),S\left(t\right),D\left(t\right)\right]$$

*L_2_input linguistic layer*: The neurons in this layer perform fuzzification. As shown in Equations (4) and (5), 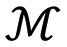
(·) is the linguistic variable related to the input value. Namely $O_{i}^{2}$ is the fuzzy membership value with respect to input metric. It reflects the degree that the input value corresponds with 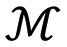
(·) (*i.e.*, the degree of $\bar{\text{RSS}}(t)$ related to linguistic variable “high” is 0.8 and the degree of *V*(*t*) related to linguistic variable “very low” is 0.2). The output of layer 2 is presented in Equation (4), where the Gaussian Function is used as the parameterized membership function. The relationship between input and output of each neuron is shown in Equation (5). Each row in matrix **M***^N^*^×^*^T^* is a linguistic variable set related to one dimension of the input state vector. Increasing the dimension of linguistic variable set will lead to a high accuracy of the reinforcement sensor, while the computational complexity will rise exponentially. Based on this consideration, we use a five dimensional linguistic variable set. Our simulations verify that this quantity of linguistic variables can guarantee the accuracy of the sensor with a low computational complexity:

(4) $$O_{i}^{2}=M^{N\times T}=\left\lceil \begin{array}{ccc} {ℳ}_{1,1}\left(u_{1}^{2}\right) & \cdots & {ℳ}_{1,T}\left(u_{1}^{2}\right)\\ \vdots & \ddots & \vdots \\ {ℳ}_{N,1}\left(u_{N}^{2}\right) & \cdots & {ℳ}_{N,T}\left(u_{N}^{2}\right) \end{array}\right\rceil$$

(5) $${ℳ}_{i,j}\left(u_{i}^{2}\right)=exp\left(-\frac{1}{2}{\left(\frac{u_{i}^{2}-m_{i,j}}{\sigma_{i,j}}\right)}^{2}\right),\forall i\in \left\{1,2,\ldots ,N\right\};\forall j\in \left\{1,2\ldots ,T\right\}$$

*L_3_rule layer*: This layer achieves the fusion of the fuzzy rules. The output is equal to the fuzzy multiplication operation for each input value. It implements the precondition of the T-S logic. According to the functions of layer 2, the continuous state space of input metrics has been divided by the membership functions into discrete sub-state spaces. *s_k_* and 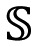
*^TN^* are the sub-state and the entire sub-state space, respectively. Therefore, 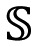 = {*s_k_*|*k* ∈ {1, 2, …, *T^N^*} }. For the *i* th dimension, the membership set of the input vector **U**^1^ (*N*) is ${\text{M}}_{i}=\left[{ℳ}_{i,1}\left(u_{i}^{2}\right),{ℳ}_{i,2}\left(u_{i}^{2}\right),\cdots ,{ℳ}_{i,T}\left(u_{i}^{2}\right)\right]$, ∀*i* ∈ {1, 2, …, *N*}, where *T* is the quantity of membership function. ℝ*^TN^*, is the rule space mapping the discrete sub-state space. Each element in ℝ*^TN^*, which is denoted by *δ_k_* (*s_k_*), can be regarded as weighting for the sub-state *s_k_*. The rule value is calculated by Equation (6), where ∀*k* ∈1, 2, …, *T^N^*}:

(6) $$\mathbb{R}=\{\delta_{k}\left(s_{k}\right)|\delta_{k}\left(s_{k}\right)=\left(\forall {ℳ}_{1,j_{1}}\left(u_{1}^{2}\right)\in {\text{M}}_{1}\right)\times \left(\forall {ℳ}_{2,j_{2}}\left(u_{2}^{2}\right)\in {\text{M}}_{2}\right)\times \cdots \times \left(\forall {ℳ}_{i,j_{N}}\left(u_{N}^{2}\right)\in {\text{M}}_{N}\right)\}$$

Equation (7) is the output of layer 3, where $u_{i,j}^{3}$ is the *j*th membership of *i*th input metric:

(7) $$\begin{array}{c} O_{k}^{3}=\delta_{k}\left(s_{k}\right)=\underset{i}{\text{∏}}u_{i,j}^{3}=\underset{i}{\text{∏}}\left(\left\{{ℳ}_{i,j_{i}}\left(u_{i}^{2}\right)|\forall {ℳ}_{i,j_{i}}\left(u_{i}^{2}\right)\in {\text{M}}_{i}\right\}\right)\\ \forall i\in \left\{1,2,\ldots ,N\right\},\forall j\in \left\{1,2,\ldots ,T\right\},\forall k\in \left\{1,2,\ldots ,T^{N}\right\} \end{array}$$

*L_4_output linguistic*: Every neuron in this layer includes a local action-reinforcement pair, which is represented as (*a_i_*, *q_i_*). The action set is a predefined finite set including the probable solutions in output space. For example with regard to a vertical handoff decider, the local action set is defined as **A** = {*a*_1_ (strongly reject), *a*_2_ (reject), *a*_3_ (access), *a*_4_ (strongly access)}. Although there are only two final handoff decisions (*i.e.*, access and reject), here we use a more detailed action set including four local actions in pursuit of higher resolution for the output linguistic space, which can enhance the accuracy of the system. Corresponding to each sub-state *s_k_*, the local action *a_i_* ∈ **A** is guided by the related local reinforcement *q*(*s_k_*, *a_i_*). Assuming the optimal local action is $a_{k}^{*}$, satisfying Equation (8):

(8) $$a_{k}^{*}=\underset{a_{i}}{\arg\max}\left\{q\left(s_{k},a_{i}\right)\right\},\forall a_{i}\in A,\forall k\in \left\{1,2,\ldots ,T^{N}\right\}$$

The output of layer 4 is the normalized local action and the local reinforcement value. They are denoted as Equations (9) and (10), respectively:

(9) $$O_{k}^{4}=\delta_{k}\left(s_{k}\right)\times a_{k}^{*}\times {\left(\sum_{i=1}^{T^{N}}\delta_{k}\left(s_{k}\right)\right)}^{-1}=O_{k}^{3}\times a_{k}^{*}\times {\left(\sum_{i=1}^{T^{N}}O_{k}^{3}\right)}^{-1}$$

(10) $${\tilde{O}}_{k}^{4}=\delta_{k}\left(s_{k}\right)\times q\left(s_{k},a_{k}^{*}\right)\times {\left(\sum_{i=1}^{T^{N}}\delta_{k}\left(s_{k}\right)\right)}^{-1}=O_{k}^{3}\times q\left(s_{k},a_{k}^{*}\right)\times {\left(\sum_{i=1}^{T^{N}}O_{k}^{3}\right)}^{-1}$$

*L_5_ defuzzy & ouput layer*: The neurons in the last layer achieve defuzzification by linear summation. The global action 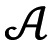 and global reinforcement ℚ) (see Definitions 1 and 2) are obtained by the fusion of the local action and local reinforcement as shown in Equations (11) and (12), respectively:

(11) $$O_{i}^{5}=\sum_{K=1}^{T^{N}}u_{i}^{5}$$

(12) $${\tilde{O}}_{i}^{5}=\sum_{K=1}^{T^{N}}{\tilde{u}}_{i}^{5}$$

***Definition 1:*** let 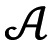 (**U**^1^(*t*)) be the global action base on the input state vector **U**^1^(*t*); then 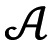
* (**U**^1^(*t*)) is the optimal global action combined by every optimal local actions $a_{k}^{*}$, which are all guided by $q(s_{k},a_{k}^{*}).$

***Definition 2:*** let 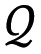 (**U**^1^(*t*), 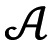
(**U**^1^(*t*))) be the global reinforcement value with respect to the state-action pair (**U**^1^(*t*), 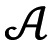
(**U**^1^(*t*))); correspondingly, 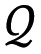
(**U**^1^(*t*), 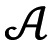
*(**U**^1^(*t*))) is the optimal global reinforcement related to the optimal global action 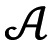
*(**U**^1^(*t*)).

Overall, the global action 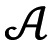 and the global reinforcement ℚ value can be represented as follows:

(13) $$A^{*}\left({\text{U}}^{1}\left(t\right)\right)=\sum_{k=1}^{T^{N}}\left(\delta_{k}\left(s_{k}\right)\times a_{k}^{*}\times {\left(\sum_{k=1}^{T^{N}}\delta_{k}\left(s_{k}\right)\right)}^{-1}\right)$$

(14) $$𝒬\left({\text{U}}^{1}(t),A^{*}\left({\text{U}}^{1}(t)\right)\right)=\sum_{k=1}^{T^{N}}\left(\delta_{k}\left(s_{k}\right)\times q\left(s_{k},a_{k}^{*}\right)\times {\left(\sum_{k=1}^{T^{N}}\delta_{k}\left(s_{k}\right)\right)}^{-1}\right)$$

#### 3.2. Backward Propagation

According to Figure 2, the backward propagation of the RFS will be triggered after an OBU switching back to the cellular network. The backward propagation phase achieves the sensing and parametric learning functions. The handoff controller evaluates the complete handoff actions involving the DVH process and UVH process and then calculates the reinforcement sensing signal according to the policies we predefine below. The parametric conclusions in layer 4 of the RFS associated with all possible combinations of the linguistic variables can be tuned by the reinforcement sensing signal.

The OBU state transition is $({\text{U}}^{1}(t),A^{*}\left({\text{U}}^{1}(t)\right))\;\underset{\to }{\text{handoff}}{\text{U}}^{1}\left(t+\tau_{l}\right)$. According to the update principle from classical Q-learning [18], the global reinforcement signal is updated by Equation (15), where α is learning rate, *β* is discount factor, 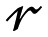 is reward sensing value, and *τ_ɭ_* is the handoff latency:

(15) $$Q^{\prime }\left({\text{U}}^{1}(t),A^{*}\left({\text{U}}^{1}(t)\right)\right)=\left(1-\alpha \right)Q\left({\text{U}}^{1}(t),A^{*}\left({\text{U}}^{1}(t)\right)\right)+\alpha \left(r+\beta Q\left({\text{U}}^{1}\left(t+\tau_{l}\right),A^{*}\left({\text{U}}^{1}\left(t+\tau_{l}\right)\right)\right)\right)$$

The difference of *Q* value is represented by Equation (16):

(16) $$\begin{array}{ll} \Delta Q & =Q^{\prime }\left({\text{U}}^{1}(t),A^{*}\left({\text{U}}^{1}(t)\right)\right)-𝒬\left({\text{U}}^{1}(t),A^{*}\left({\text{U}}^{1}(t)\right)\right)\\ & =\alpha \left(r-𝒬\left({\text{U}}^{1}(t),A^{*}\left(U^{1}(t)\right)\right)+\beta 𝒬\left({\text{U}}^{1}\left(t+\tau_{l}\right),A^{*}\left({\text{U}}^{1}\left(t+\tau_{l}\right)\right)\right)\right) \end{array}$$

According to an ordinary gradient decent principle, the local reinforcement *q* value can be updated by Equation (17):

(17) $$q^{\prime }\left(s_{k},a_{k}^{*}\right)=q\left(s_{k},a_{k}^{*}\right)+\Delta q=q\left(s_{k},a_{k}^{*}\right)+\Delta Q\times \delta_{k}\left(s_{k}\right)\times {\left(\sum_{k=1}^{T^{N}}\delta_{k}\left(s_{k}\right)\right)}^{-1}$$

The reward sensing value 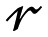 in the form of a positive or negative value is evaluated according to the validity of the handoff decision after the OBU logs out of the RSU. It is applied to tune the local *q* value. It should be set as a normalized value that reflects the system performance. As discussed in Section 2, handoff behaviors for high individual throughput should be encouraged. Positive reward sensing should therefore be used. On the other hand, if a terminal switches to a RSU and obtains a lower average individual throughput than that the cellular network, it should be given a negative reward sensing value. The absolute value of the reinforcement sensing value reflects how well the handoff decision satisfies or dissatisfies the objective. To distinguish the symbol of reward sensing from the symbol of the individual throughput, we present the former in a script style.

The *r_c_* and *r_w_*(*t*) are the average individual cellular and WLAN throughput at time instant *t*, respectively. When the OBU switches into RSU, the immediate individual throughput is lower than the cellular network. The reward sensing is given as follow, where *t_w,in_* is the time instant for the duration of handoff latency due to the association and authentication procedures.

If *r_w_* (*t_w,in_*) < *r_C_*, then the reward sensing signal is defined as:

(18) $$r=\frac{r_{W}\left(t_{W,in}\right)-r_{C}}{r_{C}}$$

Assuming an OBU is approaching a RSU, *t_w,sens_* is the timing that the OBU initiates the first handoff requests, and *t_w,out_* is the timing that the OBU initiates the procedure of UVH. To explore the feasibility of the handoff and the best handoff timing, we define the reward sensing as follows, where *r_w,avr_* is the average individual throughput during the period of (*t_w,out_*− *t_w,sens_*). The calculation for *r_w,avr_* is shown in Equation (19). The average individual throughput includes the data transmission for both cellular and WLAN:

(19) $$\begin{array}{c} r_{W,\text{avr}}=\frac{1}{t_{W,\text{out}}-t_{W,\text{sens}}+2\tau_{l}}\times \left(\int_{t_{W,\text{sens}}}^{t_{W,in}-\tau_{l}}r_{c}(t)dt+\int_{t_{W,in}}^{t_{W,\text{out}}}r_{W}(t)dt\right)\\ =\frac{1}{t_{W,\text{out}}-t_{W,\text{sens}}+2\tau_{l}}\times \left(r_{c}\left(t_{W,in}-\tau_{l}-t_{W,\text{sens}}\right)+\int_{t_{W,in}}^{t_{W,\text{out}}}r_{W}(t)dt\right) \end{array}$$

If *r_W,avr_* ≥ *r_C_*, as we discussed before, positive reward sensing is given by:

(20) $$\begin{array}{c} r=\frac{r_{W,\text{avr}}-r_{C}}{r_{W,\text{avr}}}\\ =1-r_{C}\times \left(t_{W,\text{out}}-t_{W,\text{sens}}+2\tau_{l}\right)\times {\left(r_{c}\left(t_{W,in}-\tau_{l}-t_{W,\text{sens}}\right)+\int_{t_{W,in}}^{t_{W,\text{out}}}r_{W}(t)dt\right)}^{-1} \end{array}$$

In this paper, we suppose data to be transmitted is known information. However, for the cases that data to be transmitted cannot be known from each handoff request, if *r_W,avr_* ≥ *r_C_*, several conditions need to be considered as follow:

(a) OBU does not finish data transmitting during the period of associating with RSU;
(b) OBU finishes data transmitting during the period of associating with RSU, and data to be transmitted is known information;
(c) OBU finishes data transmitting during the period of associating with RSU, and data to be transmitted is unknown information.

For conditions (a) and (b), a negative reward sensing is given by Equation (21). Note that, a particular case is presented in condition (c). A wrong handoff decision is taken, and the problem is that we do not know if the car had or not traffic to send. It should be the best decision with the information available. Trying to reject similar handoff requests afterwards is not right. Generally, condition (c) happens at a low probability (data transmitting finished immediately after handoff triggered). It is hard to evaluate the feasibility of handoff decision for this type of handoff requests. In avoid of causing the degeneration of the system performance, reward sensing is given by 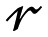 =0 for condition (c):

(21) $$\begin{array}{c} r=\frac{r_{W,\text{avr}}-r_{C}}{r_{C}}\\ \;=\frac{1}{r_{C}\left(t_{W,\text{out}}-t_{W,\text{sens}}+2\tau_{l}\right)}\times \left(r_{c}\left(t_{W,in}-\tau_{l}-t_{W,\text{sens}}\right)+\int_{t_{W,in}}^{t_{W,\text{out}}}r_{W}\left(t\right)dt\right)-1 \end{array}$$

Here we demonstrate an example to explain the working schemes for Equations (18)–(21). If a vehicle switches to a WLAN but then stops exchanging traffic immediately, the proposed mechanism will judge the reasonability of the handoff behavior according to the parameter *r_w,avr_* in Equation (19). Because the OBU suffers from handoff latency, but benefits little from WLAN, it is likely to be an unnecessary handoff, which is not encouraged. If *r_W,avr_* < *r_c_*, the OBU achieves lower average individual throughput after executing the handoff than if it stayed in the cellular network. The reward sensing value is calculated by Equation (21). A negative reward 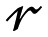, which also can be recognized as a punishment signal, is obtained and used to tune the reinforcement system. For the adaptive learning capability, the proposed mechanism will remember this handoff behavior, and try to reject similar handoff requests thereafter.

### 4. Performance Evaluation

A typical outdoor vehicular heterogeneous wireless communication scenario covered by cellular and WLAN is shown in Figure 1. We assume RSUs are deployed at the road side with 400 m inter-distance and are 10 m away from the roadway. They operate at 5.9 GHz and adopt the same PHY as IEEE 802.11a, except that only 10 MHz channel spacing is used instead of 20 MHz, for the reason of the increased RMS delay spread in the vehicular environments. Due to the 10 MHz channel spacing, two-folded timing parameters provide longer guard interval to offset increased RMS delay, and therefore, preventing inter-symbol interference. [22]. Depending on MCS level, the WLAN can provide data rate ranging from 3 Mbps to 27 Mbps including signaling cost. The Distributed Coordination Function (DCF) with binary exponential backoff algorithm is adopted in MAC layer, and the related packet transmitting parameters are demonstrated in Table 2. As discussed in Section 2, we suppose MIB attribute is set to “false”, so that vertical handoff behavior always followed by a relatively considerable latency.

The simulation is based on MATLAB. For WLAN, the link level parameters can be found in [1]. We assume that the OBU can be tuned at an optimal MCS level according to its RSS at any time. The saturated throughput and achievable individual throughput can then be calculated according to [23]. The CDMA2000 1x-EV network [24] supplies the global coverage. We simply suppose the bandwidth of each link of the cellular network is 0.6 Mbps. The service types we include are non-real-time and non-safety related applications (e.g., file transmission and non-real time streaming). The initial data bits need to be transmitted following a negative exponential distribution with an expectation of 200 Mb. The applications benefit from high data rate, while they are not sensitive to delay and jitter. The vehicles arrive at the coordinates [0,0] m and initially link with cellular network. The arrival rate ranges from 0.3 veh/s to 0.5 veh/s. The coverage of WLAN is relatively small; therefore, we assume that during the period that vehicles move into the coverage area, they keep a uniform linear motion state with velocities ranging from 20 km/h to 70 km/h. A typical log-normal function is used as the propagation model for the WLAN signal, as shown in Equation (22):

(22) $$P\left(d\right)=P_{t}-P_{o}-10\gamma lg\left(d\right)+\varepsilon \left(\mu ,\sigma \right)$$

**Figure 4.**
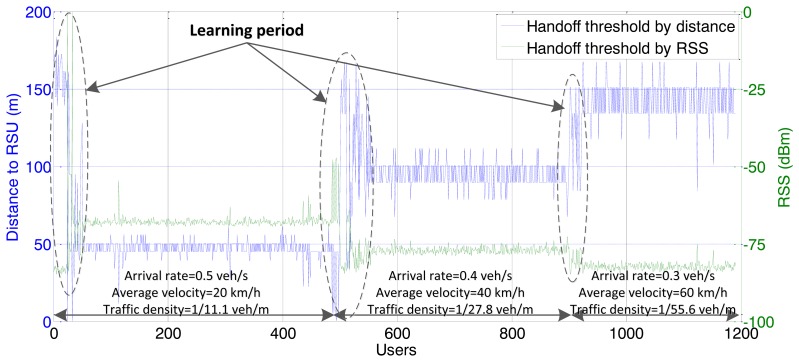
Vertical handoff threshold *vs.* different traffic conditions.

**Figure 5.**
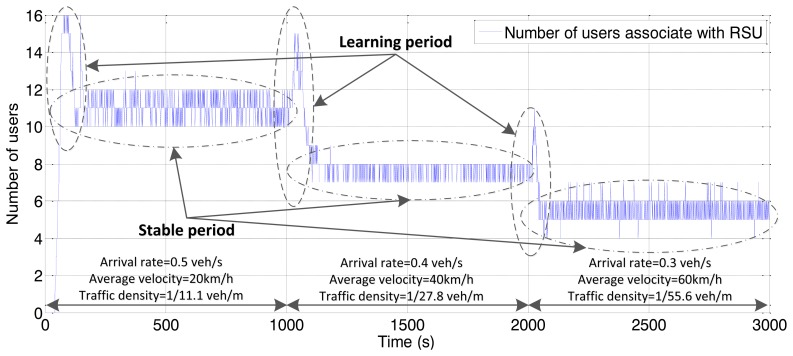
Quantity of users associated with RSU *vs.* different traffic conditions.

For shared channel (uplink and downlink) of the IEEE 802.11, the simulations involve the consideration of both downlink traffic and uplink traffic. The simulation results for throughput are the values of downlink plus uplink. We first investigate the performance of the proposed handoff controller, based on vehicle velocity and traffic density. As shown in Figures 4 and 5, the simulation consists of three stages with different parameters for arrival rate and vehicle velocity, which lead to different traffic densities.

The simulation time in each stage is 1,000 s. We switch the parameter setting to another group when the simulation time is finished. Due to a lack of *a priori* knowledge added to the RFS, the handoff threshold appears as a stochastic trend at the beginning of each stage, which is depicted as the “learning period” in Figures 4 and 5. With more simulation loops, the RFS is tuned by collecting more and more knowledge from the sensing signal to produce a stable trend. The improved performance validates the adaptive sensing and learning ability of the proposed algorithm.

As observed in the simulation results, when traffic density is relatively high, the RFS controller tends to compress the accessing area to avoid too many OBUs, which may result in the degeneration of the overall network performance. This policy only enables the potential handoff initialized by OBUs with relatively higher RSS, and it can enhance the channel utility for heavy traffic loads. The upper bound of the vehicle quantity associated with the RSU is 12 according to from Figure 5. This means that additional terminal cannot achieve better individual throughput from WLAN than the cellular network. As the traffic load becomes lighter, the controller enlarges the accessing area. The threshold for the accessing radius reaches approximately 150 at the third stage. However the number of users declines as the traffic becomes sparse. Shadow fading causes the threshold to appear as an oscillatory trend. This is more obvious in the form of distance.

**Figure 6.**
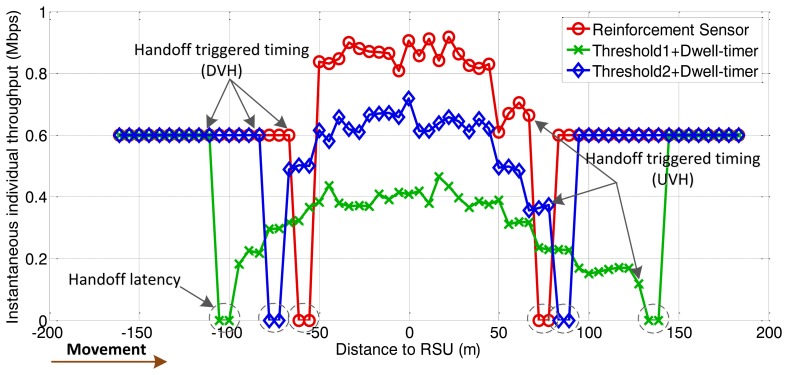
Instantaneous individual throughput *vs.* distance to RSU (arrival rate = 0.5 veh/s).

**Figure 7.**
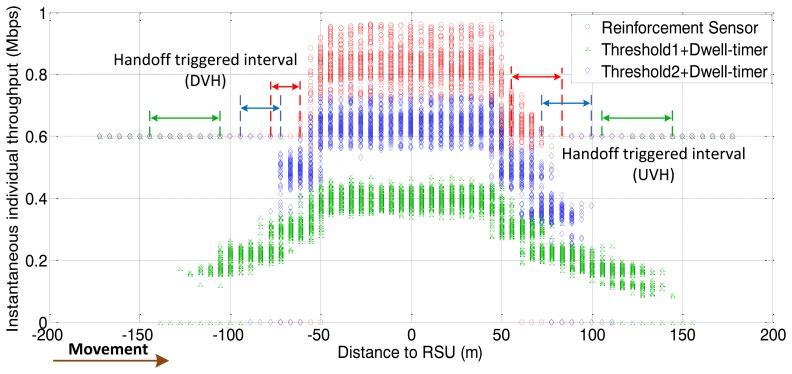
Distribution of individual throughput *vs.* distance to RSU (arrival rate = 0.5 veh/s).

**Figure 8.**
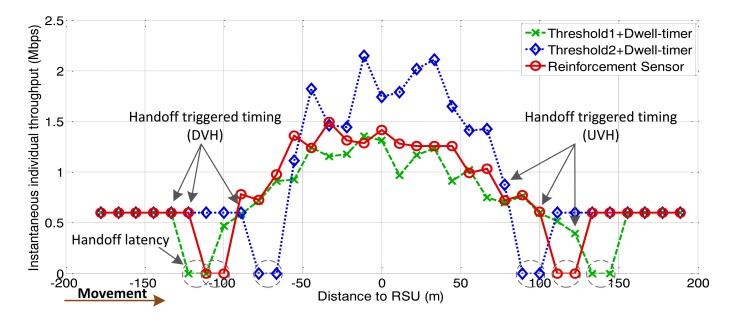
Instantaneous individual throughput *vs.* distance to RSU (arrival rate = 0.4 veh/s).

**Figure 9.**
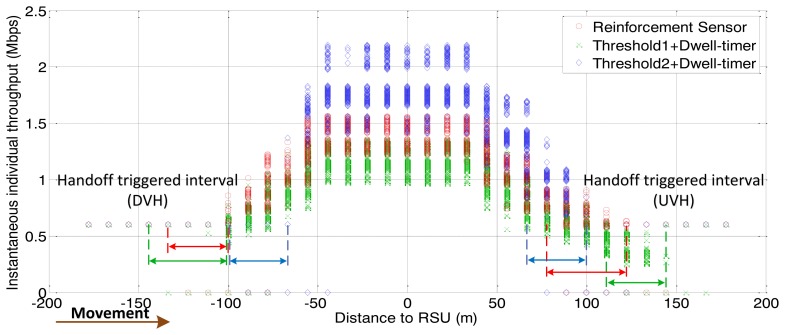
Distribution of individual throughput *vs.* distance to RSU (arrival rate = 0.4 veh/s).

**Figure 10.**
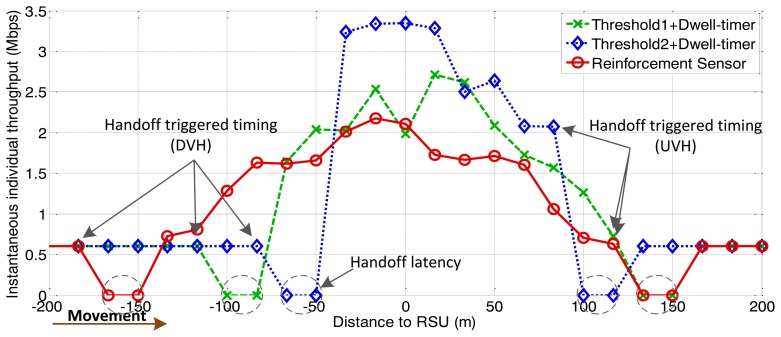
Instantaneous individual throughput *vs.* distance to RSU (arrival rate = 0.3 veh/s).

**Figure 11.**
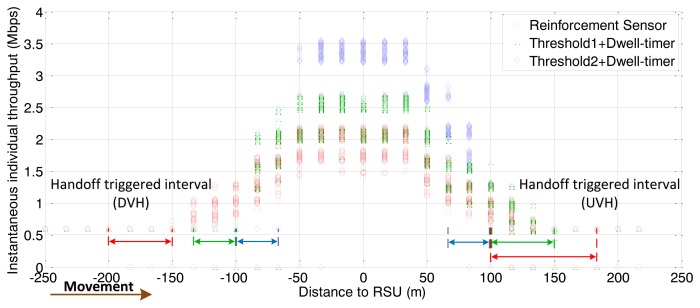
Distribution of individual throughput *vs.* distance to RSU (arrival rate = 0.3 veh/s).

The following figures demonstrate the performance with respect to the individual throughput of vehicles. Figures 6 and 7 are based on the condition of the first stage in Figure 4. Figures 8 and 9 are based on the condition of the second stage in Figure 4. Figures 10 and 11 are based on the condition of the last stage in Figure 4, respectively. We use two conventional fixed thresholds plus a dwell-timer based handoff controller for comparison. Thresholds 1 and 2 are set to the sensitivity of the lowest MCS level plus 5 dB and 10 dB, respectively. The dwell-timer is set to 2 s. In Figures 6, 8 and 10, we randomly select one vehicle from the simulation results, and present its handoff process for each of the three schemes, describing the individual throughput *versus* the distance to the RSU. The vehicle approaches the RSU and then moves away. During this period, there are many other OBUs simultaneously accessing the same RSU. Figures 6, 8 and 10 are expected to give an overall expression about the system performance by different vertical handoff strategies. Figures 7, 9 and 11 show the simulation results for 100 samples, describing the approximate distribution of individual throughput. The simulation results in these figures are randomly sampled from the stable period shown in Figure 5. In these figures, vehicles move from the left side to the right and periodically exchange handoff signaling with the RSU. They initially link with the cellular network and implement DVH and UVH according to the vertical handoff controller. The vertical handoff process is considered as a hard handoff. The OBUs have to cut off the link with the original network when the handoff is triggered. Therefore, the handoff latency, which achieves association and authentication, results in a “gap” of individual throughput during each handoff process. According to the distribution of these “gap” values, the interval for the handoff can be inferred.

In Figure 6, the proposed algorithm applies a higher access threshold than the other two algorithms for high traffic density. As a result, vehicles within approximately 70 m of the RSU are permitted to access the service. Compared with the proposed algorithm, the other two algorithms cannot guarantee better throughput performance for WLAN under heavy traffic conditions. The algorithm represented by the green curve reflects the fact that, the individual throughput provided by WLAN is lower than by the cellular network due to a heavy network load. There has already been a degeneration of the overall network performance. From the statistical simulation results in Figure 7, the times at which the handoffs are triggered are appropriate. The data rate for WLAN is just higher than that of the cellular network when vehicles finish the DVH process. As the vehicles depart from the RSU, they are switched back to the cellular network when WLAN cannot supply a better data rate. This keeps the terminals connected to the best network.

Compared with the first condition, the traffic density is sparser in the two scenarios shown in Figures 8, 9, 10 and 11. The RFS enlarges the valid coverage area to permit more vehicles and longer service time. This is based on the premise that WLAN guarantees a better individual throughput performance.

From Figure 9 we can observe that the algorithm represent by the green markers adopts large accessing area. However, the individual throughput for WLAN is not always better than the cellular network. This means that vehicles implement the DVH process too “early”. Correspondingly, the UVH process is initiated at a later time than it should be. Compared with the performance achieved by the algorithm represented by the blue markers in Figure 9, the RFS based handoff controller provides a larger valid coverage. Figure 11 demonstrates that, for light traffic loads, the RFS extends the valid coverage area as large as possible to permit more users and longer service time.

**Figure 12.**
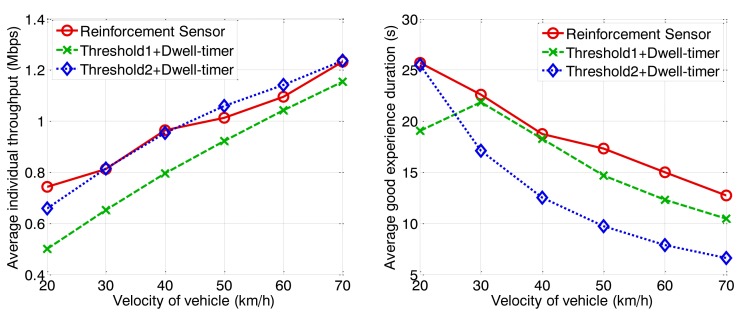
Average individual throughput and good experience duration *vs.* velocity of vehicle (arrival rate = 0.5 veh/s).

**Figure 13.**
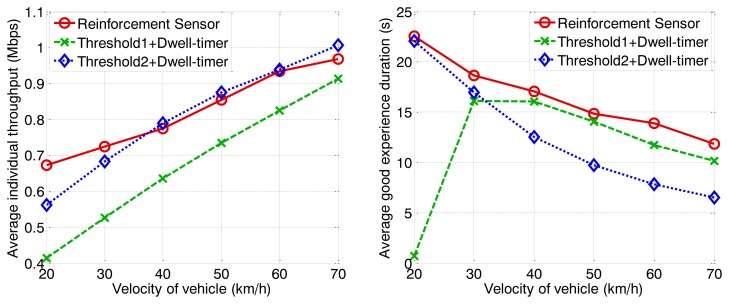
Average individual throughput and good experience duration *vs.* velocity of vehicle (arrival rate = 0.4 veh/s).

**Figure 14.**
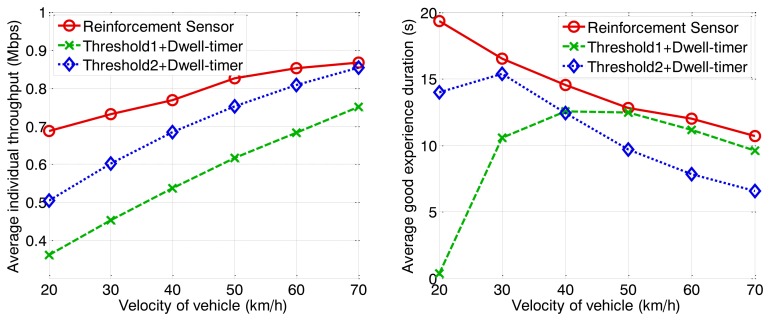
Average individual throughput and good experience duration *vs.* velocity of vehicle (arrival rate = 0.3 veh/s).

To investigate the QoE, we define a parameter named good experience duration. It is the time period that the vehicle achieves a better individual throughput for WLAN than for the cellular network. This parameter can reflect the effectiveness of the handoff control algorithm. The other parameter we discuss is the average individual throughput. This is the average data rate that users achieve from the heterogeneous wireless network without a signaling cost. The simulation results are shown in Figures 12, 13 and 14 with arrival rates of 0.3, 0.4, and 0.5 vehicles per second, respectively.

With increasing vehicle velocity, the average individual throughput increases, because the high velocity results in a sparse traffic density according to a given arrival rate, leading to a reduction in user quantity. Fewer associated terminals means that there is higher saturated throughput due to the MAC scheme for IEEE 802.11p. In contrast to the individual throughput, good experience duration decreases because the dwelling time for WLAN coverage becomes shorter. The proposed handoff controller can guarantee an average individual throughput higher than 0.6 Mbps under various conditions. However, the other two algorithms cannot match this characteristic for high traffic loads, where vehicles arrive with a low average velocity but high an arrival rate. The proposed scheme can always execute the handoff behavior at an appropriate time instant, to ensure a better data rate for WLAN than for the cellular network. Under the high traffic loads depicted in Figure 14, the proposed algorithm has major advantageous. The algorithm represented by the blue curve achieves a better average individual throughput than the proposed algorithm when the traffic is sparse because a high threshold is adopted. As a result, the valid accessing area is small and relatively few users are permitted to access the WLAN. The channel utility is high because of a low packet collision probability. However, potential duration of vehicle association with the RSU is very short, correspondingly. This leads to good experience duration, demonstrated by the low blue curves in Figures 12, 13 and 14, are quite low. Besides that, we can observe the good experience duration represented by the green curve is the lowest when traffic load is heavy because the handoff threshold is too low for the given condition. The inappropriate handoff decisions result in the degeneration of network performance. In summary, the proposed algorithm can adjust the handoff control strategy for different conditions to ensure that RSUs work optimally, thereby guaranteeing high QoE for users.

### 5. Conclusions

In this paper, we propose a reinforcement sensor embedded vertical handoff control strategy to support mobility management in vehicular heterogeneous wireless networks. The proposed algorithm has a real-time learning capability and can adaptively provide optimal handoff decisions to realize the Always Best Connected concept. It integrates vehicular mobility, traffic load, handoff latency, network status, and link level data transmitting conditions. Moreover, prior knowledge such as the channel parameter is not a necessity for the handoff controller due to its sensing and learning ability. This advantage can save substantial human and financial resources for measurement in practical applications. We focus on a heterogeneous scenario consisting of a global cellular network complemented by a vehicle to infrastructure communication mode. Simulation results verify that, the proposed algorithm can provide optimal handoff decisions to keep terminals always connected with the best network under different conditions. Furthermore, it can ensure that road side units work appropriately and can guarantee a high quality user experience.

## Figures and Tables

**Table 1.** Pseudo-code for the proposed handoff control scheme.

**Procedure at OBU**
**if** communicating with cellular network **and** an RSU is detected
Send beacon frame periodically with terminal measures $\left\{\bar{\text{RSS}}(t),V(t),D(t)\right\}$
**elseif** communicating with RSU
Send beacon frame periodically with terminal measures RSS (*t*)
**endif**
**Procedure at RSU** (loop by handoff control interval)

**DVH control for new user *i***
Receive terminal measures $\left\{\bar{\text{RSS}}(t),V(t),D(t)\right\}$
Send measures $\left\{\bar{\text{RSS}}(t),V(t),D(t)\right\}$ to RFS
Handoff control according to the output of RFS
**UVH control for associated user *j***
Receive terminal measures *RSS* (*t*)
Judge the trend of WLAN signal according to FFT-DD
Handoff control according to the output of FFT-DD and threshold
Calculate reward sensing value 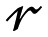
Update reinforcement sensing signal *q*

**Table 2.** Simulation parameters.

***Parameter***	***Value***
Tx power of RSU (***P****_t_*)	100 mW
Path loss in the first meter (***P*_0_**)	37.3 dB
Path loss exponent (**γ**)	3
Gaussian shadow fading (***μ, σ***)	(0,5) dB
Handoff latency (***τ****_ɭ_*)	2 s
Beacon interval	50 ms
Data rate in cellular network (***r****_c_*)	0.6 Mbps
Vehicle's initial coordinate	[0,0]
RSUs' coordinates	[400,10] m, [800,10] m,…
Vehicle's velocity (***V***)	20–70 km/h
Vehicle's arrival rate	0.3–0.5 veh/s
Average traffic density	11.1 m/veh–64.8 m/veh
Data bits required to be sent (**λ**)	200 Mb
Handoff control interval	1 s
Channel spacing	10 MHz
RSS sensitivity of different MCS level	[−85, −84, −82, −80, −77, −73, −69, −68] dBm
Saturate data rate at different MCS level	[3,4.5,6,9,12,18,24,27] Mbps
MAC layer	IEEE 802.11 DCF
Minimum backoff window size (***W***)	16
Maximum window size (**2***^m^****W***)	64
Time duration of short interframe space	28 μs
Time duration of distributed interframe space	130 μs
Slot time	51 μs
PHY header	128 bits
MAC header	272 bits
Packet Payload	8,184 bits
ACK	240 bits
